# The ninth life of the cat reference genome, Felis_catus

**DOI:** 10.1371/journal.pgen.1009045

**Published:** 2020-10-22

**Authors:** Wengang Zhang, Jeffrey J. Schoenebeck

**Affiliations:** The Roslin Institute and Royal (Dick) School for Veterinary Studies, University of Edinburgh, Easter Bush, Midlothian, United Kingdom; HudsonAlpha Institute for Biotechnology, UNITED STATES

**Fig 1:**
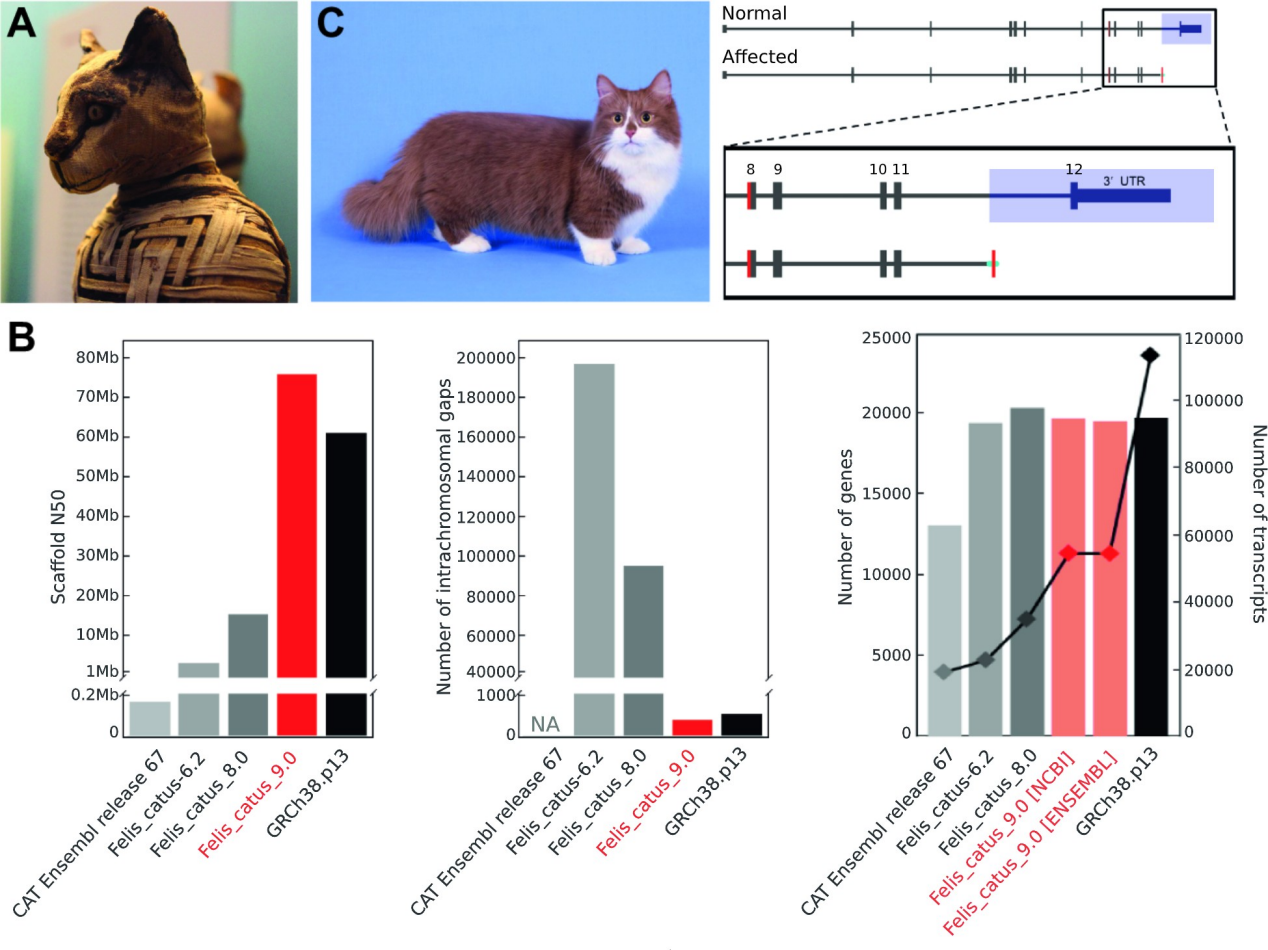
A summary of Felis_catus_9.0. (A) Egyptian mummified cat from the Roman period, housed at The British Museum. (B) Comparison of assemblies. GRCh38.p13 is the reference genome of *Homo sapiens*. (C) Asymmetric dwarfism observed in Munchkin breed cats is likely due to a 3.3 kb deletion in the *UGDH* gene [5]. *UGDH*, UDP-glucose 6-dehydrogenase. *Image credit*: *Justin Ennis*, *Flickr*.

Few animal species are as storied and intertwined with human history as domestic cats, *Felis catus*. With an estimated 600 million cats living with humans, cats’ popularity as pets is indisputable. The earliest hint of our relationship with felines come from Neolithic skeletal remains found in Cyprus, where a human and wildcat were co-interred some 9,500 years ago [1]. Five thousand years later, the human–cat bond would be proclaimed in ancient Egyptian iconography and burials (Fig 1A), as well as through the mitochondrial DNA of many contemporary cats whose mitotypes were traced back to Northern Africa. The dispersal of cats from Anatolia, the Levante, and Northern Africa coincided with human trade and agriculture [2,3]. Though companionship was probably welcome, it is predation of rodent pests that likely precipitated the union between man and cat [4].

Buckley and colleagues present new insight into the domestic cat genome and genetic variation in this issue of *PLOS Genetics* with the release of Felis_catus_9.0, the newest version of the cat reference genome [5]. With its dramatic improvements in both assembly contiguity and annotation, this reference represents a significant step forward for scientists and cat lovers interested in demography, evolution, domestication, and genomic medicine in companion animals.

Contemporary household cats share both our environment and our exposures to infectious diseases. As they grow old, many will succumb to age-related diseases whose names ring familiar: diabetes, lymphoma, kidney disease, cardiomyopathy, and dementia to name a few. For many cats, these morbidities are managed with varying degrees of success through access to state-of-the-art veterinary care. As companion animals, dogs also share many of these same attributes; however, the 2 species’ genetic architectures are distinct: unlike most dogs that can be categorized to various degrees as belonging to a breed, the vast majority of cats are the products of random mating. As a result, the genetic composition of “random breds,” by far the most common type of household cat worldwide, is comparatively diverse and not uncommonly admixed with local subspecies of wildcats [1,6,7]. From this perspective, the genomic architecture of the cat is more akin to our own in terms of diversity and population stratification. For these reasons, cats have the potential to be a comparative model of human medicine and disease etiology.

In 2006, the International Cat Genome Sequencing Consortium (ICGSC) released ASM18133v3, an assembly produced from a female Abyssinian named “Cinnamon.” This draft assembly was produced from Sanger-sequenced plasmid and fosmid libraries. At just 2× read coverage, the assembly was highly fragmented: only 50% of the genome was covered by contigs of lengths greater than 2.7 kb (a statistic also known as contig N50). It required 174,000 contigs to cover half the cat genome [8], and genome assembly and annotation depended heavily on radiation hybrid and comparative mapping to human and dog genomes.

The ICGSC has incrementally improved Cinnamon’s reference genome through releases rebranded as Felis_catus. Felis_catus_5.0 and Felis_catus_6.2 incorporated read data from bacterial artificial chromosome (BAC) end sequencing (2× coverage), 454 Titanium GS_FLX (12× coverage), and enhanced scaffolding, which was underpinned by improved radiation hybrid maps [9,10], together with some variant and methylation data [11]. In 2014, Felis_catus_6.2 was supplanted by Felis_catus_8.0, which added 20× pooled Illumina short-read sequencing from wildcats and pedigree cats to vastly increase variant information. Scaffolding was also improved through yet another high-density radiation hybrid map [12]. However, the Felis_catus assembly remained stubbornly fragmented, more than 80× as much as its fellow carnivore, the dog, whose assembly benefitted from deeper Sanger sequencing (and capital) at the outset.

With the advent of long-read sequencing and optical mapping technologies, the ICGCS were among the first to apply them to mammalian genomes. Their efforts, embodied by Felis_catus_9.0, were released to the wider research community in November 2017. Low-passage fibroblasts grown from Cinnamon provided high molecular weight DNA that was key to maximizing the benefits of Pacific Biosystem’s long-read sequencing technology and Bionano Saphyr’s single-molecule optical mapping technology. This quality of this chromosome level assembly rivaled other popular species including human (Fig 1B), mouse, rat, pig, cattle, and goat.

As described by Buckley and colleagues, Felis_catus_9.0 is remarkably contiguous, with 4,909 contigs and an N50 of 42 Mb and exceptionally long gap-free segments (Fig 1B). The improved assembly facilitated production of gene models, a process whose ab initio predictions were refined by RNA-sequencing, which was used to profile numerous tissues. Improvements in genomic features also include the definition of noncoding genes, pseudogenes, and novel genes that were absent in previous versions of Felis_catus.

Buckley and colleagues also describe approximately 40,000,000 single-nucleotide variants (SNVs) and approximately 13,000,000 indels based on resequencing data from 74 animals. Beyond using annotation tools to predict the functional impact of these variants, the authors binned these variants by functional constraint: significantly fewer loss of function mutations were observed in genes with essential functions. Rather, constrained genes were differentially enriched for presumably benign synonymous variants. This examination, novel in its application to nonhuman species, provides additional granularity to uncovering disease-causing alleles. The authors also generated a comprehensive structure variation (SV) atlas. Over 200,000 SVs (insertions, deletions, inversions, and duplications) were identified, a 300-fold increase from Felis_catus_6.2. One of the SVs was discovered in resequenced Munchkins. It occurs on chromosome B1, within a 5.2 Mb region that was previously associated with this breed’s disproportionate dwarfism [13]. The putatively causal allele removes the final exon of UDP-glucose 6-dehydrogenase (*UGDH*), a gene whose product is postulated to participate in proteoglycan synthesis within the articular cartilages of long bones (Fig 1C).

The improvements made to the cat reference genome are likely to yield many more biological insights that will impact medicine across many mammalian species. However, Felis_catus_9.0 is but one of many steps that are needed to make genomic veterinary medicine common practice. Significant hurdles remain in the quest to deliver genomic medicine to cats and other companion animals: annotations that depict RNA isoforms are sparse; National Center for Biotechnology Information (NCBI) and ENSEMBL gene models often conflict; noncoding epigenetic features are lacking; Y chromosome assemblies are incomplete [14,15]; and the sequence contents of nucleolar organizer regions, telomeres, and centromeres are undefined. As more conspecific assemblies are produced [16], how will we integrate their information?

Companion animal cell lines and cell-based assays are needed to functionally validate variants suspected of causing disease. Clearly, future research investments and technological innovations are needed to create the repertoire of resources that are required to ensure that clinical DNA samples are interrogated exhaustively and interpreted correctly. Not to be overlooked, the costs of clinical sequencing are currently too high for most owners, especially in the absence of health insurance.

The dream of bringing genomic medicine to veterinary clinics must overcome these and other formidable challenges; scaling these challenges will require research team initiatives, project coordination, sustainable funding, and most importantly, communicating to funders and science policy makers the unmet need that our community can fill. To the latter point, we must unapologetically embrace and communicate that cats (and dogs) are more than pets; they are also unique clinical animal models and sentinels of human health.
